# The rise of New Guinea and the fall of Neogene global temperatures

**DOI:** 10.1073/pnas.2306492120

**Published:** 2023-09-25

**Authors:** Peter E. Martin, Francis A. Macdonald, Nadine McQuarrie, Rebecca M. Flowers, Pierre J. Y. Maffre

**Affiliations:** ^a^Department of Geological Sciences, University of Colorado, Boulder, CO 80309; ^b^Department of Earth Science, University of California, Santa Barbara, CA 93106; ^c^Department of Geology and Environmental Science, University of Pittsburgh, Pittsburgh, PA 15260; ^d^Department of Earth and Planetary Science, University of California, Berkeley, CA 94720; ^e^Aix-Marseille Université, CNRS, Institut de Recherche et Développement (IRD), Institut National de Recherche pour l'Agriculture, l'Alimentation et l'Environnement (INRAE), Collège de France, Centre de Recherche et d'Enseignement en Géosciences et Environnement (CEREGE), 13545 Aix-en-Provence, France

**Keywords:** silicate weathering, ophiolites, thermochronology, New Guinea, Miocene

## Abstract

The ~2,000-km-long Central Range of New Guinea is a hotspot of modern carbon sequestration due to the chemical weathering of igneous rocks with steep topography in the warm wet tropics. These high mountains formed in a collision between the Australian plate and ophiolite-bearing volcanic arc terranes, but poor resolution of the uplift and exhumation history has precluded assessments of the impact on global climate change. Here, we develop a palinspastic reconstruction of the Central Range orogen with existing surface geological constraints and seismic data to generate time–temperature paths and estimate volumes of eroded material. New (U-Th)/He thermochronology data reveal rapid uplift and regional denudation between 10 and 6 Mya. Erosion fluxes from the palinspastic reconstruction, calibrated for time with the thermochronological data, were used as input to a coupled global climate and weathering model. This model estimates 0.6 to 1.2 °C of cooling associated with the Late Miocene rise of New Guinea due to increased silicate weathering alone, and this CO_2_ sink continues to the present. Our data and modeling experiments support the hypothesis that tropical arc-continent collision and the rise of New Guinea contributed to Neogene cooling due to increased silicate weathering.

## Significance

The Earth’s climate has cooled by 5 to 10 °C over the past 15 My, but it is unknown whether this interval of geological climate change is due predominantly to a decrease in CO_2_ sources through volcanic outgassing or an increase in global weatherability and CO_2_ sinks. New thermochronology data and a coupled weathering-climate model estimate that mountain building in New Guinea from 10 to 6 Ma increased carbon sinks and consumed the CO_2_ equivalent of 0.6 to 1.2 °C, contributing to Neogene global cooling.

Since the Miocene Climatic Optimum (~17 to 14 Ma) when sea surface temperatures (SST) and ocean bottom waters were 5 to 10 °C warmer than today (1–4), the Earth’s climate has cooled, with an acceleration of SST cooling after 10 Ma to near modern temperatures by ~6 Ma (5, 6). The drivers of this Late Miocene cooling trend are poorly understood, but the magnitude and timescale of the shift indicate changes to the geological sources and/or sinks of carbon (6, 7). Recently, it was suggested that a decrease in midocean ridge spreading rate led to a decline in volcanic CO_2_ outgassing sources and contributed to Neogene cooling (5, 8). Alternatively, it has been proposed that cooling was associated with arc-continent collision in the tropics and subaerial emergence of the SE Asian islands (Maritime continent), due to an increase in CO_2_ sinks associated with the chemical weatherability of silicate rocks (9–11).

Chemical weatherability, or surface reactivity (12), is set by temperature, runoff, relief, and lithology (13). Mafic and ultramafic lithologies are both more Ca- and Mg-rich and more soluble, thereby consuming more CO_2_ during chemical weathering than their felsic counterparts and having outsized leverage as a CO_2_ sink (14–16). These components of weatherability are maximized during arc-continent collision in the warm, wet tropics, which has inspired the hypothesis that the emplacement of large (ultra)mafic-rich ophiolites and concomitant creation of steep topography at low latitudes has contributed to Phanerozoic cooling trends (10, 17, 18).

**Fig. 1.**
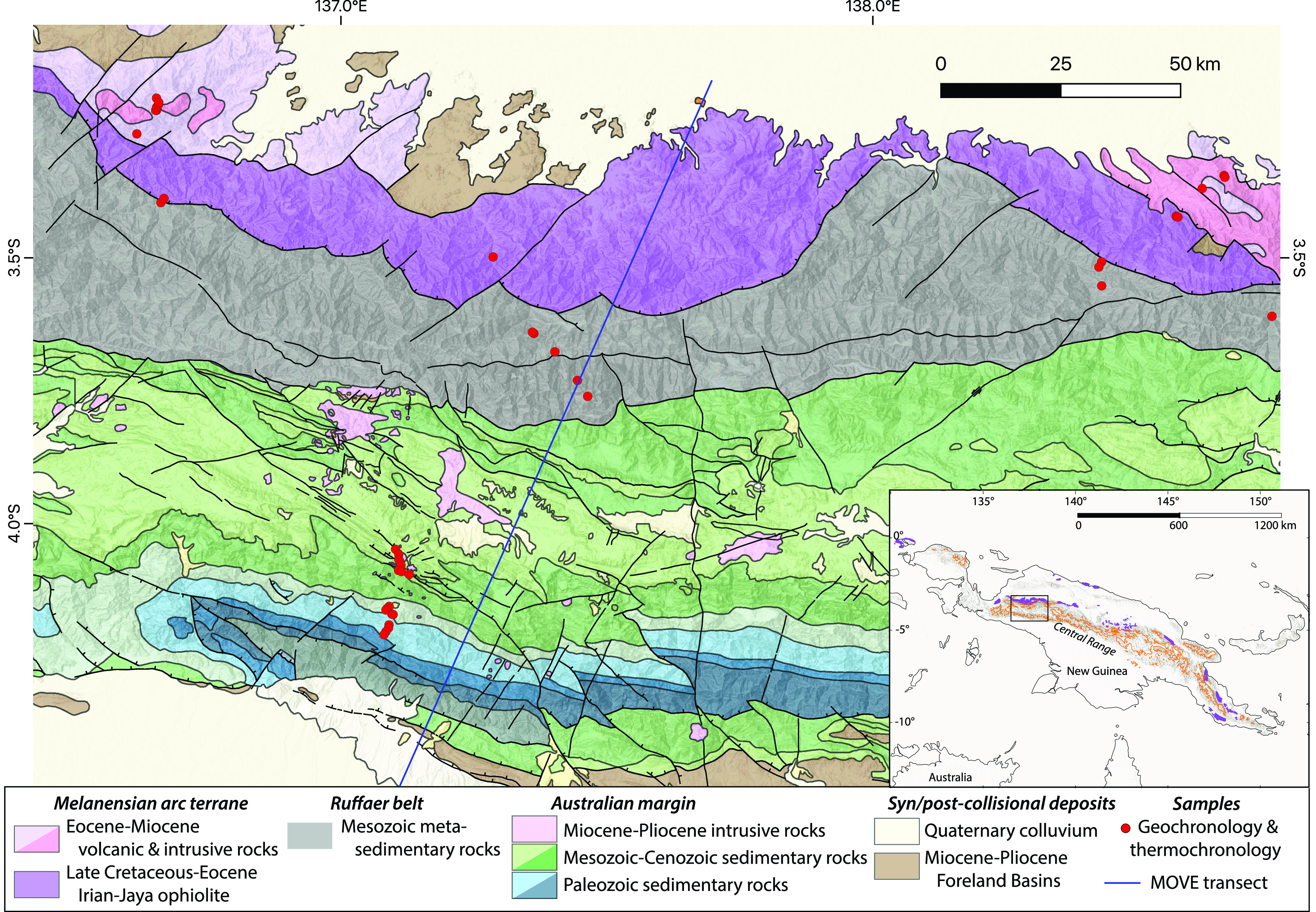
Geologic map of the study area in Papua, Indonesia, on the island of New Guinea. Geological map data courtesy of Freeport-McMoRan-Indonesia. AHe (apatite (U-Th)/He) and ZHe (zircon (U-Th)/He) data acquired in this study. AFT data reported in refs. 20 and 21. Purple areas in the *Inset* are ultramafic bodies, and the orange is a 3-km topographic contour, showing the high topography of the island.

New Guinea is the largest SE Asian island and hosts the greatest mountain range in the Maritime continent, the Central Range, which is ~2,000 km long with peaks over 5 km high, comparable in size to the Alps (Fig. 1). It is also the site of the most recent arc-continent collision in the tropics, in which one of the world’s largest ophiolites was tectonically emplaced onto thick continental crust, creating high topography with increased orographic precipitation (19, 20). Thus, the hypothesis that tropical arc-continent collisions have contributed to global climate change in the geological record predicts that the tropical exhumation of the Irian Jaya ophiolite should be associated with a specific global cooling trend (10). To test this hypothesis, we use low-temperature thermochronology, a palinspastic reconstruction of the kinematic evolution of this region, and thermokinematic modeling to constrain the topographic and erosional history of the New Guinea arc-continent collision. We then employ the weathering component of the GEOCLIM Earth system model (9) to estimate the impact of New Guinea uplift and erosion on global climate.

## Geological Background

The area of subaerially exposed land in SE Asia increased from the Miocene to present associated with the ongoing arc-continent collision between Australia and the Sunda-Banda arc system (9, 22, 23). The Central Range formed through the collision of the Melanesian arc with the Australian continent above a north dipping slab (19, 24–26). The initial Miocene collision was followed by the Pliocene collision of the Finisterre terrane in eastern New Guinea and establishment of the modern strike-slip boundary (27). Prior to collision with the thick Australian continental crust, the Melanesian arc terranes likely formed more subdued topography similar to the Mariana Islands or Tonga.

In the Central Range, the geology can be divided into four main lithotectonic belts: Tertiary sedimentary basins (the Meervlakte fore-arc basin to the north and the New Guinea foreland basin to the south), the Irian Jaya ophiolite and associated arc volcanics (including the Auwewa volcanics in the west and the Dabera complex to the east), the Ruffaer metamorphic belt, and the Irian fold-and-thrust belt (Fig. 1). The highest elevations occur in the fold-and-thrust belt and exceed 5,000 m.

Previous work on the metamorphic belt in the Central Range has emphasized the enormous amount of total exhumation (>20 km in some locations) (20). However, it has remained unclear precisely when and where the ophiolite was exhumed and major topography was generated. Existing fission-track data provide a first-order constraint on the timing of uplift and erosional exhumation in several areas across New Guinea (20, 21, 28–30). Fission-track studies on the southern flank of the range suggested that exhumation was largely younger than 3 Ma (20), but stratigraphic constraints indicate some erosion of the ophiolite by Middle to Late Miocene (31). Due to the evolution of fault motion in space and time, uplift and exhumation are not evenly distributed across the island; for example, Apatite fission-track (AFT) dates suggest that cooling rates vary by over a factor of five in the Irian fold-and-thrust belt (20). In spite of these variable rates, a consistent pattern of rapid cooling (and therefore exhumation) between 20 and 3 Ma is recorded by these data in multiple locations across the island (20, 21, 28–30).

The exhumation history of the New Guinea ophiolites is particularly poorly constrained, and the mapping of their areal extent varies considerably within the literature (20, 30, 32). The Marum ophiolite in the eastern half of the Central Range (33), the third largest contiguous ophiolite body on New Guinea, yielded a single AFT age of 5.1 ± 1.5 Ma (30), indicating early Pliocene exhumation. In our study area, the Irian Jaya ophiolite (the largest ophiolite on New Guinea) is juxtaposed with the Ruffaer metamorphic belt on the southern margin of the ophiolite (Fig. 1), indicating variable unroofing rates across strike (21). In this region, episodes of exhumation have been proposed at ~12 Ma and 4 Ma from clustering of AFT dates from the metamorphic and igneous rocks (21). Initial uplift of the Central Range at ~12 Ma is supported by the occurrence of Mesozoic macrofossils (ammonites and belemnites), metapelitic rock fragments, ultramafic rocks and igneous rock clasts in the Middle to Upper Miocene (16 to 8.5 Ma) Makats Formation in the Meervlakte forearc basin directly north of the Central Range (31), as well as by the cessation of carbonate shelf sedimentation around this time (26). Weiland and Cloos (20) inferred that uplift and exhumation subsequently accelerated at ~4 Ma to present. However, prior to this study, the existing AFT data had not yet been quantitatively integrated with thermochronologic and other data to generate an exhumation model for the orogen.

## Erosion Magnitude and Flux

New and existing thermochronologic data were gathered from a total of 40 samples across the three main lithotectonic belts in the study area: the Irian Jaya ophiolite, the Ruffaer pelitic schist metamorphic belt, and the Irian fold-and-thrust belt (Fig. 1). Samples located in the ophiolite and associated volcanic units yield AHe dates from 7 to 3 Ma, AFT dates from 12.3 to 8 Ma, and ZHe dates that generally decrease from 16 to 8 Ma southward toward the metamorphic-ophiolite unit boundary (Fig. 1). The Ruffaer metamorphic and Irian fold-and-thrust belt samples have generally younger dates than the ophiolite, with nearly all chronometers returning indistinguishable ages of <3.5 Ma, except for a single sample near the unit boundary with older ZHe dates (Dataset S1). These data broadly indicate rapid late Neogene rock cooling throughout the study area, with the most rapid/recent rock cooling occurring in the fold-and-thrust and metamorphic belts.

A challenge of interpreting erosion and topographic uplift in collisional orogens from the thermochronology data alone is that compressional forces may be accommodated by variable fault motions, which include rock uplift, translation, and burial during thrusting. For this reason, a palinspastic reconstruction of the Central Range orogeny (blue line on Fig. 1) was developed using the software Move (Petex) that replicates modern geologic observations (e.g., topography, lithology, and structure) and generates viable paths of rocks toward the surface (Movies S1 and S2) that can be directly compared to the measured thermochronometric data. Within this kinematic context, thermochronometers are sensitive to both the vertical and lateral components of motion, with dates that cluster in time representing focused erosional exhumation due to vertical fault motion, and gaps between dates potentially indicating times of primarily lateral motion.

The palinspastic reconstruction was imported into the thermokinematic model Pecube-D to evaluate predicted time-temperature (tT)-paths and compare modeled versus measured dates (34–36). Pecube calculates heat production and heat transport via advection and diffusion according to input thermal parameters. We use a modified version of this model, Pecube-D (37–39), where advection of material is defined by the deformed 2D grid of points generated during the construction of the palinspastic cross-section, with timesteps defined by an input velocity field. The simulated thermal histories combined with mineral diffusion kinetics are used to predict the AHe, AFT, and ZHe dates at the surface for each model step.

**Fig. 2.**
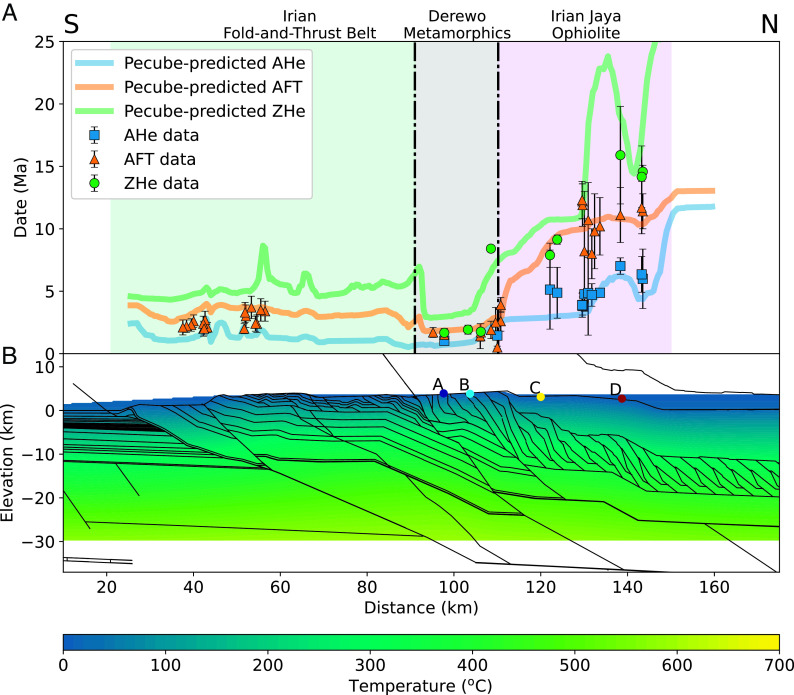
Central Range orogeny reconstruction. (*A*) Pecube-predicted thermochronometer dates with actual data overlain. Predicted dates are shown only where sedimentation has not occurred (*SI Appendix*, Fig. S2). Data from the (U-Th)/He method are shown as the mean and 1 SD of replicate analyses (N ≥ 3); fission-track data are the pooled age and 1σ uncertainty derived from counting statistics and zeta calibration error. (*B*) The final step of the Move model with the Pecube-calculated temperature field (no vertical exaggeration). Colored dots are the final positions of particles depicted in the temperature–time plots shown in Fig. 3.

A structural, erosional, topographic, and surface uplift history of the Central Range orogeny is derived from the above approach (Fig. 2 and *SI Appendix*, Fig. S2 and Movies S1 and S2). The observed dates, which are fully independent of the thermokinematic modeling, were projected onto the cross-section for comparison with model predictions (Fig. 2*A*). Shortening velocities were iteratively adjusted to reduce the residuals between predicted and observed dates until a close fit was observed. Such a fit is not necessarily possible. If a model is fundamentally incompatible with the data, no set of adjustments to shortening velocity or thermal parameters would yield a pattern of predicted dates that replicate the observations. The close fit between model predictions, thermochronometric data, and geologic information (Fig. 2*A*) is consistent with the reconstruction (Fig. 2*B*) and indicates that the modeled magnitude and rates of exhumation are valid.

**Fig. 3.**
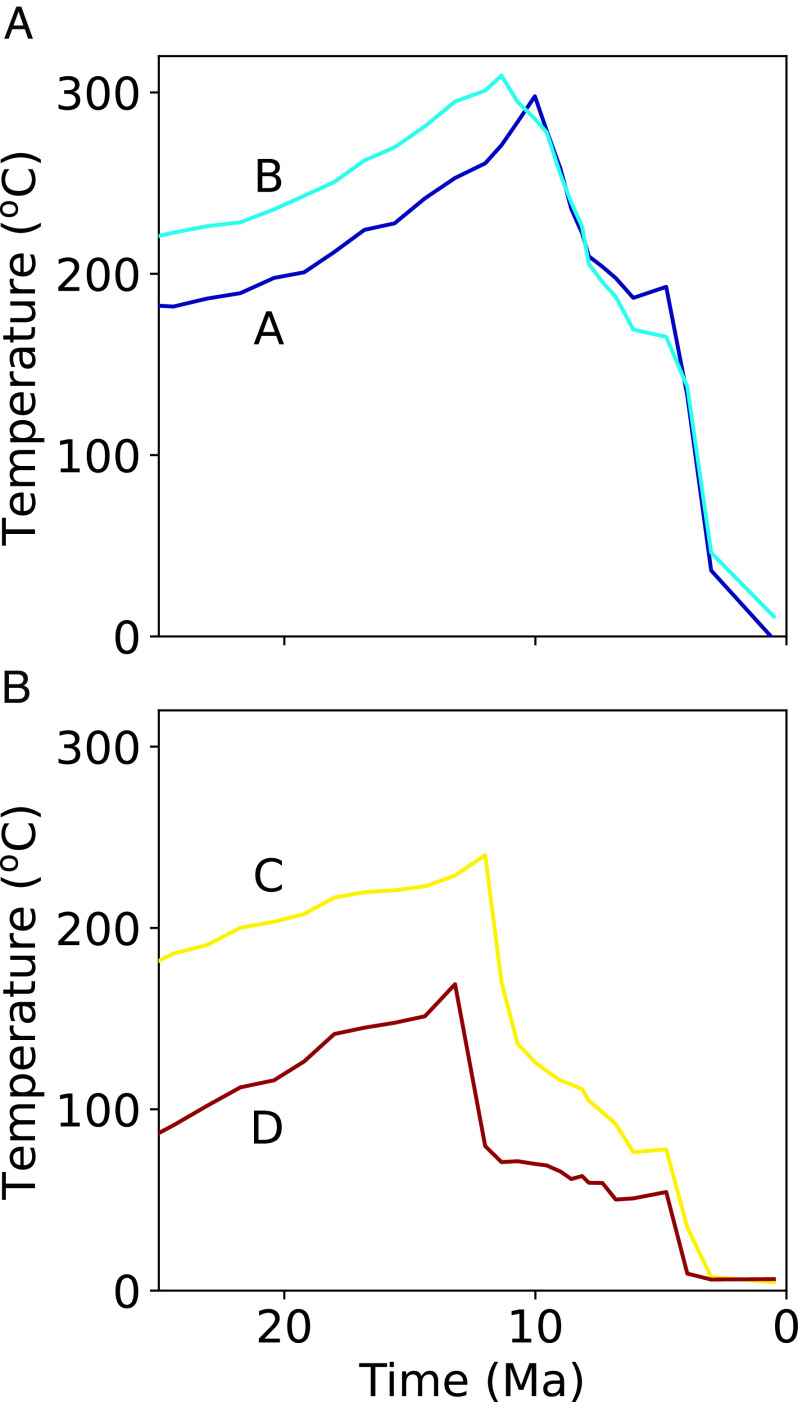
Temperature–time plot of particle paths from selected positions near samples from (*A*) the metamorphic belt and (*B*) the ophiolite. Exhumation occurred in each path during the cooling trend. The thermal trajectory is generated in the thermokinematic model with input from the palinspastic reconstruction (see *Materials and Methods* and Movies S1 and S2). Time is refined with the thermochronological data provided herein. Locations of particles are shown in Fig. 2 and *SI Appendix*, Fig. S2. Note that the ophiolite was thrust over the metamorphic belt in (*A*) and was being eroded while the metamorphic belt was being exhumed (*SI Appendix*, Fig. S2 and Movies S1 and S2).

The thermal history of individual particle paths (Fig. 3) is provided by the Pecube model from the input of the cross-section kinematics that replicates the existing geological data and the assumed subsurface thermal field. Boundary conditions, evaluated thermophysical parameters, and other model parameters used are discussed in *Materials and Methods* and provided in *SI Appendix*, Tables S3 and S4. The sensitivity of Pecube model results to these parameters and variations in cross-section geometry and kinematics have been reviewed extensively (40–44). The thermochronology dates for the samples are compared with the dates predicted by the thermal histories at the sample locations in the Pecube model and thus add the time dimension to the model (Fig. 3). The comparison of modeled to measured dates is used to evaluate the exhumation modeled in Move and refine the shortening velocities, but the thermochronology data are not an input into the thermokinematic model at any point. This workflow has been successfully applied to reconstruct the shortening and exhumation histories, and replicate cooling histories, of several other mountain belts (36, 40–46).

**Fig. 4.**
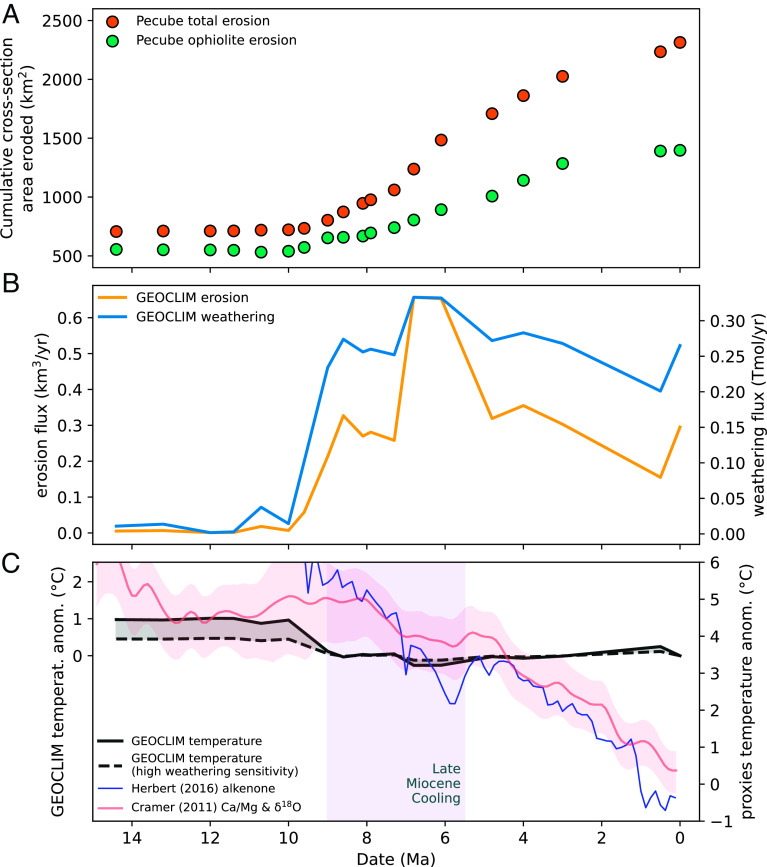
Erosion history of the Central Range orogeny and predictions for weathering flux and climate cooling. (*A*) The cumulative history of all erosion and ophiolite-only erosion along the cross-sectional reconstruction in Fig. 2. (*B*) Erosion and weathering fluxes calculated by the GEOCLIM model for the whole island of New Guinea. (*C*) Estimation of global mean temperature anomaly due to weathering flux in (*B*) using the high and low values of weathering sensitivity (*SI Appendix*, Fig. S5) compared to temperature proxies from alkenone-derived sea surface temperature average of all latitudes (6) and Mg/Ca and sea-level corrected benthic oxygen isotope data (3).

Cross-sectional erosion magnitudes and fluxes from the Central Range were calculated from the palinspastic reconstruction (Fig. 4*A*), calibrated for time with the thermochronological data (*Materials and Methods*). In GEOCLIM, erosion is proportional to slope, so for each time step, we uniformly multiplied the modern slope field by the cross-sectional model-derived (Move and Pecube) erosion flux of the current time step divided by the final (i.e., “modern”) model-derived erosion flux (Fig. 4*B*). The corresponding chemical weathering fluxes were then calculated by GEOCLIM with runoff and temperature fields from the ERA5 climate reanalysis (47).

The approach described above assumes a uniform change in slope through time along the length of the Central Range, which is supported by the continuity of topography and lithotectonic units (Fig. 1). Although significant tectonic complexities occur on the eastern and western margins of the Central Range (24, 48), the Bird’s Head and Bird’s Tail regions are relatively small and have a negligible impact on the GEOCLIM calculations.

## Weathering Sensitivity

To keep the carbon cycle balanced on the timescale of carbon in the ocean/atmosphere system, a local increase in weathering must be compensated by a reduction in weathering elsewhere. In the period of transition to this new steady state, global weathering rates exceed geologic CO_2_ input rates (from volcanism or oxidative weathering of organic carbon) and atmospheric CO_2_ levels fall (7). Global cooling associated with this weathering increase can be estimated from multiple global GEOCLIM simulations coupled with climate models at variable CO_2_ concentrations to determine temperature sensitivity to weathering. The weathering sensitivity is distinct from a direct climate sensitivity relationship between CO_2_ concentration and temperature.

Using the Geophysical Fluid Dynamics Laboratory (GFDL) CM2.0 climate model (49) GEOCLIM simulations indicate a quasi-linear scaling of 1 °C of global cooling per 0.26 Tmol/y of local weathering increase, regardless of the CO_2_ change needed to cool the Earth by 1 °C (*SI Appendix*, Fig. S5). However, another set of GEOCLIM simulations using Community Earth System Model (CESM) 1.2.2.1 with a slab ocean model shows a nonlinear evolution, ranging from 0.27 to 0.56 Tmol/y/°C (50). The functional form of the weathering-global temperature relationship remains a significant source of uncertainty. In an attempt to quantify it, we use two values of weathering sensitivity, derived from these additional experiments: a “low” value of 0.26 Tmol/y/°C and a “high” value of 0.56 Tmol/y/°C (*SI Appendix*, Fig. S5).

In GEOCLIM, the Southeast Asian islands currently account for 11.5 ± 1.2% (*Materials and Methods*) of global chemical weathering flux, of which New Guinea contributes 44 ± 4% at 0.27 Tmol/y (~5% of the global flux). The GEOCLIM-calculated erosion and weathering fluxes rapidly increase to ~0.3 km^3^/y and ~0.26 Tmol/y, respectively, between 10 and 8.5 Ma (Fig. 4*B*), followed by a rise in erosion rate to ~0.65 km^3^/y between 7 and 6 Ma with a less pronounced rise in silicate weathering to ~0.33 Tmol/y. Together, this corresponds to a total cooling of ~1.2 °C in the 10 to 6 Ma period in the low weathering sensitivity case or ~0.6 °C in the high weathering sensitivity case (Fig. 4*C*).

The erosion and weathering fluxes calculated by GEOCLIM are not perfectly correlated because of the nonlinearity between slope and chemical weathering (calibrated with modern observed fluxes, ref. 9) and imply that the ranges of New Guinea are currently close to the kinetically limited weathering mode (*SI Appendix*, Fig. S6). Any additional increase in the erosion rate therefore may not serve to increase weatherability but could have provided a mineral surface shuttle for increased organic carbon burial (51).

## Discussion

The results presented here suggest that ophiolite exhumation and the generation of high mountain topography in New Guinea first occurred between 10 and 8.5 Ma when Australia and New Guinea were at a latitude of ~10°S drifting northward into the modern intertropical convergence zone (9). This history can account for 0.6 to 1.2 °C of global climate cooling from 10 to 6 Ma. Estimates of sea surface temperature (SST) from 10 Ma to present from the alkenone proxy (5, 6), clumped oxygen isotopes (1), and Mg/Ca and sea-level corrected benthic oxygen isotope data (2, 3) suggest 4 to 6 °C of cooling from 10 to 5.5 Ma such that accelerated chemical weathering in New Guinea alone may account for 10 to 33% of this cooling trend (Fig. 4*C*).

The timing of climate cooling yielded by the model is slightly older and predicts a more abrupt temperature decline than observed in the SST data (Fig. 4*C*). Several factors can account for this slight offset between model predictions and observations, including changes in circulation, other orogenic events in SE Asia, and concomitant changes in organic carbon burial (51). For example, the GEOCLIM experiment assumes modern latitude and circulation patterns. If rainfall was lower when New Guinea was at 10°S, then the increase in global weatherability and the inferred 0.6 to 1.2 °C of cooling may have been more gradual between 10 and 6 Ma, after which time New Guinea was deep into the tropics. In addition, after Late Miocene cooling, the northward drift of New Guinea further modified oceanic and atmospheric circulation as the Indonesian through-flow became more restricted by 3.5 Ma (52).

Our study focused on the topographic growth of the Central Range of New Guinea, but other paleogeographic changes in SE Asia likely contributed to an increase in carbon sinks. Importantly, Timor was exhumed in the Pliocene to Present associated with the collision of the Banda arc (53). Additionally, New Guinea grew outward after it grew upward, with the exposed land area north of the Central Range increasing over the past 5 Ma (9, 23), whereas the GEOCLIM weathering flux simulation assumes modern area (Fig. 3*B*). This growth of subaerially exposed land includes the Coastal Range of New Guinea, which was exhumed from the Pliocene to Present due to the collision of the Finisterre terrane (27). A progressive increase in exposed land area in this region would have induced a more gradual increase in CO_2_ consumption via chemical weathering than assumed in our model, particularly because the Central Range is in the kinetically limited weathering regime where eroded sediments from the high relief areas are progressively chemically weathered in larger catchment systems with longer residence times (*SI Appendix*, Fig. S6).

The hypothesis that the growth of SE Asian islands contributed to Neogene cooling has been portrayed as inconsistent with the coeval rise in seawater ^87^Sr/^86^Sr (54). This model used an unradiogenic ^87^Sr/^86^Sr value of 0.7045 for riverine input from these islands (54); however, compiled rock compositions of SE Asian islands have an average ^87^Sr/^86^Sr value of 0.7085 (11). Our exhumation model predicts that approximately half of the eroded material from New Guinea was sourced from the unradiogenic ophiolite and half from other sources with more radiogenic ^87^Sr/^86^Sr compositions, including the metamorphic belt (Fig. 4*A*). This is consistent with riverine sediments from the Sepik River, on the northern flank of the Central Range, which and have an average ^87^Sr/^86^Sr value of 0.7097 in clay and 0.7065 in silt (55). Using a more moderate ^87^Sr/^86^Sr input value of ~0.7085, an increased riverine flux from the SE Asian islands can reproduce the seawater ^87^Sr/^86^Sr curve (11), and does not preclude Neogene cooling through the emergence of the SE Asian islands (56).

We show that the development of the largest mountain belt in the tropics could have exerted an outsized role in global cooling through accelerated CO_2_ consumption from intensified chemical weathering. Although a Neogene decrease in midocean ridge spreading rates may have been associated with a decline in volcanic CO_2_ outgassing and long-term climate cooling (5), we estimate that tropical arc-continent collision and the rise of New Guinea contributed 0.6 to 1.2 °C cooling between 10 and 6 Ma due to increased silicate weathering alone, even without considering additional climate cooling contributions from this orogen due to changes in organic carbon burial, modified oceanic and atmospheric circulation, and other paleogeographic changes in SE Asia. The establishment of the New Guinea CO_2_ sink helped push the Earth past the threshold for the expansion of northern hemisphere ice. The modern hotspots of chemical weathering and carbon sequestration are geologically young, and understanding their birth and development are critical for reconstructing the drivers of long-term climate change.

## Materials and Methods

Existing AFT data were combined with newly acquired apatite and zircon (U-Th)/He (AHe, ZHe) data, as well as new zircon U-Pb data, to better resolve the cooling history of the region. To evaluate the magnitude of exhumation and its evolution in space and time, we developed a palinspastic reconstruction of New Guinea from estimates of the geometry of subsurface stratigraphy and faults proposed by Hill, Keetley, Kendrick, and Sutriyono (57). We then used the thermokinematic model Pecube to predict thermochronologic dates from this reconstruction to compare with the observed thermochronologic dataset. The coupled climate-weathering model GEOCLIM was used to link the Move-derived magnitude of erosion and Pecube-derived timing of erosion with the global cooling effects of weathering associated with this erosion pattern.

All samples discussed in this paper were collected during prior studies in the province of Papua (formerly Irian Jaya), Indonesia (20, 21). Samples were collected between 1990 and 1995 in a series of five transects oriented roughly perpendicular to strike of the Central Range (of which samples from four transects are included in this work; Fig. 1). The first of these sampling transects was conducted in 1990 and 1991 across the Irian fold-and-thrust belt on the south side of the Central Range, along road cuts leading up to and within the Gunung Bijih Mining District near Puncak Jaya (the “Southern” transect; *SI Appendix*, Table S2). The latter sampling transects were conducted in 1993, 1994, and 1995 via helicopter along the north slope of the Central Range (*SI Appendix*, Table S1). The samples from these transects are described in detail by Weiland (21). Three of the sampling transects cross the contact between the Ruffaer Metamorphic Belt and the Irian Jaya ophiolite and are spread ~240 km along strike.

The sampling transect down the southern flank of the Central Range, across the Irian fold-and-thrust belt includes 17 samples from which AFT data were collected (20). From the sampling transects across the northern flank of the Central Range, apatite and zircons mineral separates were dated from the western (9 samples), central (6 samples), and eastern (8 samples) transects, for a total of 10 samples from the Ruffaer Metamorphic Belt and 13 samples from the Irian ophiolite with associated igneous units. The western transect primarily sampled the Auwewa volcanics. The eastern transect primarily sampled the Dabera intrusive complex. These two transects each extend ~2 km into the Ruffaer metamorphic belt. The central transect primarily sampled the Irian Ophiolite and the Ruffaer Metamorphic Belt.

Existing mineral separates from the samples discussed above (*SI Appendix*, Tables S1 and S2) were used to produce new (U-Th)/He apatite and zircon ages (Dataset S1). Additional mineral separates were prepared from of the samples discussed above to produce new U-Pb zircon ages (Dataset S2). The full dataset used for comparison with the Pecube model dates is reported in Dataset S3.

### Low-Temperature Thermochronology.

Because the thermal history of a given sample may be interpreted as its exhumation history, determining the time-temperature (tT) paths of a sample suite can yield detailed information about the erosion, uplift, and tectonic history of a region. Low-temperature thermochronology is based on the diffusive loss of radioactive decay products from a mineral of interest (e.g., apatite or zircon) at high temperature, while these products are retained at lower temperatures. A measurement of the ratio between the decay product and the parent nuclide thereby yields a date that is a proxy for the thermal history. Decay products can be quantitatively retained, partially lost, or fully lost over geologic time depending on the temperature path. This behavior has the effect that a range of thermal histories are permitted for a single date. Here, we apply two forms of thermochronology: fission-track and (U-Th)/He dating.

### Fission-Track Thermochronology.

The existing apatite fission track ages cited in this paper were conducted at the University of Texas at Austin. In brief, mineral separates were generated by crushing, sieving, and processing with a Rogers table, Frantz magnetic separator, and heavy liquid extraction. These mineral separates were mounted in epoxy, polished, and etched. Ages were measured using the external detector method (58) and calculated using zeta calibration methods (59). Further details regarding the analytical methods used to produce the fission track dates cited in this paper are documented in Weiland and Cloos (20) and Weiland (21).

### U-Th/He Thermochronology.

In (U-Th)/He dating, the alpha decay of ^238^U, ^235^U, ^232^Th, and ^147^Sm produces ^4^He, which is lost from individual grains by thermal diffusion at high temperatures. Helium will be quantitatively lost from zircon at temperatures ≳180 °C and from apatite at ≳70 °C. The exact temperature sensitivity of these minerals is dependent on grain size (60) and radiation damage (61–63). For young samples with relatively simple, rapid cooling histories, the kinetic effects from grain size and radiation damage are minimal (64). The long stopping distances of alpha particles during their generation cause some ^4^He to be lost from apatite and zircon of typical grain size. Here, we assume homogeneous parent nuclide distributions and apply standard alpha ejection corrections following the approach of Ketcham, Gautheron, and Tassan-Got (65) to account for the ^4^He lost from the crystal owing to ejection.

The new (U-Th)/He dates reported in this paper (Datasets S1 and S3) were produced at the University of Colorado Thermochronology Research and Instrumentation Laboratory (CU TRaIL). Apatite and zircon grains were picked based on size, crystal form, and lack of inclusions under cross-polarized light. These grains were measured, photographed, and placed in HCl-cleaned Nb packets. Helium analysis was performed using an ASI AlphaChron by heating samples with a 915-nm diode laser at 6 A for 5 min (apatite) or 15 A for 10 min (zircon). A second extraction following the same protocols was performed to ensure complete degassing of each sample. The volatiles released due to heating were cleaned using a system of scrubbers and getters to yield pure ^4^He which was spiked with a known quantity of He gas with a standard ^4^He/^3^He ratio for quantification on a Prisma Balzers quadrupole mass spectrometer.

Following He extraction, samples were retrieved from the helium line and dissolved for parent nuclide quantification. All samples were spiked with ^235^U and ^230^Th for quantification of ^238^U and ^232^Th, respectively. Apatite was additionally spiked with ^145^Nd for ^147^Sm determination. Apatite was dissolved in HNO_3_ for 2 h at 80 °C and zircon was dissolved in HF and HCl over several days in high-pressure vessels. The resulting solutions were analyzed on an Agilent 7900 ICP-MS.

Due to the mafic lithology and young exhumation timing of these samples, many of the analyzed grains were unusually small, or contained too little ^4^He and/or parent nuclide for rigorous quantification. All analyses falling below the following thresholds were removed from the dataset: F_T_ < 0.5, eU < 5× blank levels, 4He < 3× blank levels, or, for apatite crystals, initial extraction <90% of total 4He. In interpreting this dataset we exclude from the thermokinematic modeling samples with fewer than three replicate measurements and those that were not replicated within uncertainty, along with (U-Th)/He data that are clearly magmatic (rather than cooling) ages based on comparison with U-Pb data from the same unit. These excluded samples are noted in Dataset S3 and are not included in Figs. 1 and 2 of the main text. The uncertainties on the sample (U-Th)/He dates are reported as the 1 s sample SD.

### U-Pb Geochronology.

The U-Pb zircon ages from igneous rocks reported in this paper (Dataset S2) were produced at the Laser-Ablation Split-Stream facility at UC Santa Barbara following the methods described in Kylander-Clark, Hacker, and Cottle (66). Zircon was separated using standard magnetic and density techniques, mounted on an epoxy puck, polished, and imaged using the cathodoluminescence detector on UCSB’s FEI Quanta 400F field emission source SEM prior to analysis. Samples were ablated with a Photon Machines Excite 193 nm excimer laser using a spot size and repetition rate of 24 µm and 4 Hz. U-Pb isotopes were measured with a Nu Instruments Plasma HR multicollector ICPMS. The reference materials employed yielded ages within 2% of their expected values. Between 15 and 93 zircon grains were dated from each sample. Isotope data were reduced using Iolite, and mean ages were calculated with IsoplotR (*SI Appendix*, Fig. S1, 67).

Of the nine dated samples, four are from tonalites and quartz-diorites of the Dabera complex (East in Fig. 1), and five are from diorites within what is mapped as the Auwewa volcanics (West in Fig. 1). The four samples from the Dabera complex yielded ^238^U-^206^Pb ages of 29.6 ± 0.3 Ma, 27.5 ± 0.1 Ma, 27.1 ± 0.2 Ma, and 23.6 ± 0.1 Ma, recording magmatic activity in the Melanesian arc prior to collision. The five samples from the Auwewa volcanics all yielded ages between 12.7 and 12.3 Ma (*SI Appendix*, Fig. S1). This magmatism occurred around the beginning of the main collisional phase and the tectonic setting of this magmatism is uncertain. Zircon in the Auwewa volcanics have ZHe dates within uncertainty of the U-Pb dates. Because the ZHe dates do not record the regional cooling history that is the objective of our Pecube modeling, these data are excluded both from the plot in Fig. 2 and from our comparison of observed and predicted thermochronometer dates. No U-Pb dating was performed in the clastic sedimentary and metamorphic units of the Ruffaer metamorphic or Irian fold-and-thrust belts.

### Balanced Cross-Section Restoration.

The first-order geologic observations that we aim to replicate with the palinspastic model are the northern extent of the New Guinea margin (Fig. 1) the width ~150 km, depth 3 to 6 km, and age (~15 Ma to present) of the Meervlatke Basin (31, 68, 69), the age (~12 Ma to present) and depth (6 to 7 km) of the foreland basin (19, 26), the northern extent of Australian continental crust (70), the mapped surface geology (57), and the topography. We base our palinspastic reconstruction of New Guinea from estimates of the geometry of subsurface stratigraphy and faults inferred by Hill, Keetley, Kendrick, and Sutriyono (57). These authors compiled seismic, borehole, and field data to argue that the deep subsurface of New Guinea involves inverted basement extensional faults while the proximal subsurface is composed of asymmetric folds in sedimentary rocks deposited on the Australian continent. The Hill, Keetley, Kendrick, and Sutriyono (57) section ends at the Ruffaer metamorphic belt comprised of folded and faulted pelitic schist (21). Remote sensing image interpretation of this zone highlights steeply dipping south-vergent thrusting (71). We simplify these deformed rocks into a duplex structure that repeats a ~4 km thick section, based on analogous sections in New Guinea and spacing of mapped faults (71, 72), of an intermediate to distal passive margin sedimentary sequence deposited on an extended continental margin. The original thickness of the Irian Jaya Ophiolite is unknown; we base our modeled 15-km-thick ophiolite on the Semail ophiolite (73) and the burial necessary to metamorphose the Ruffaer belt.

The modified cross-section from Hill, Keetley, Kendrick, and Sutriyono (57) was overlaid with a 0.5 × 0.5 km grid of unique points and iteratively deformed using Move (Petex) in a series of ~5 to 20 km increments of displacement, with ~20 km increments during the initial 389 km of shortening associated with ophiolite emplacement and repetition of the metamorphic rocks, and 5 to 10 km increments during the final 113 km of shortening of the Australian margin. After each shortening step, the flexural isostatic thrust load was calculated from the difference between the deformed topography and previously undeformed topographic surfaces. Load density during ophiolite emplacement was 2,700 kg/m^3^ and load density of continental margin rocks was 2,500 kg/m^3^. The new topographic surface following each deformation step replicates the preexisting surface with two exceptions: 1) basins that had subsided below 0 km were assumed to have filled with sediment, and 2) areas exceeding a maximum of 4.5 km and/or slope of ~3 to 6°, based on modern observations of topographic gradients in New Guinea and early collisional islands in the western Pacific, were removed by erosion (39, 40). The effective elastic thickness (EET) used in flexural modeling was systematically varied during multiple model iterations to achieve the best balance between replicating the preserved geology at the surface and matching basin depths. The resulting EET during ophiolite emplacement was 70 km and increased to 75 km during continental margin deformation.

### Pecube-D and Thermokinematic Modeling.

The sequentially deformed grids were used to produce vectors of displacement at each grid point that become converted to velocity fields by assigning time to each deformation step. The imported velocity field allows Pecube-D (37) to calculate an evolving subsurface thermal field and produce a tT history for every point in the model. These tT paths are then used to predict dates for the thermochronometers of interest for comparison with the measured data. The thermochronometer dates were predicted using the He diffusion kinetics of Farley (74) and Reiners, Spell, Nicolescu, and Zanetti (75) for AHe and ZHe dates, respectively, and the annealing model of Ketcham, Carter, Donelick, Barbarand and Hurford (76) for AFT dates. The young age and rapid cooling histories of these samples suggest that radiation damage should have little effect on the He diffusion kinetics of apatite and zircon in these samples such that assuming kinetic models without radiation damage included is a reasonable simplification. The subsurface thermal field is sensitive to thermal parameters such as thermal conductivity (2.5 Wm^−1^K^−1^), heat capacity (800 JKg^−1^K^−1^), radiogenic heat production (3.0 µWm^3^), and e-folding depth of crustal heat production (20 km). The boundary conditions, evaluated thermophysical parameters, and other model parameters used are provided in *SI Appendix*, Table S3. An initial steady-state solution was calculated using these parameters 25 Ma prior to the start of deformation (at 35 Ma). We tested the model sensitivity to a range of e-folding depths and crustal heat production values considered typical for the rocks exposed in the region (*SI Appendix*, Table S3, 77, 78).

The model takes into account the heating and cooling of rocks due to thrust motion that places warmer hangingwall rocks over cooler footwall rocks (79). The thermal effect of this process is most pronounced where faults are deep (>15 km) and exhibit high shortening rates (>15 mm/y) (79, 80). This process is most relevant during the initial modeling stages where the ophiolite is emplaced on distal margin sedimentary rocks (35 to 20 Ma) (Movies S1 and S2). This deformation is earlier and hotter than our data allow us to rigorously assess. The 16 to 11 Ma cooling of ophiolite rocks from 250 to 75 °C (Fig. 3*B*) and the 10 to 6 Ma cooling of the Ruffaer metamorphic rocks from 250 to 75 °C (Fig. 3*A*) resulted from structural uplift due to motion on faults 5 to 15 km deeper than measured sample locations leading to surface uplift of topography and accompanying erosional exhumation. The duplexing of metamorphic rocks (~11 to 10 Ma), where sample locations are the closest to the active fault (*SI Appendix*, Fig. S2 *A* and *B*), resulted in cooling from 320 °C to 290 °C due to thrust motion and accompanying erosion. Our samples are all located kilometers from any known major faults, whereas thermal effects from fault friction have been demonstrated to occur within meters of faults (81). Similarly, thermal models of thrust faults with tens of kilometers of displacement do not show disruption of isotherms across faults (82, 83). Moreover, the narrow clustering of ages 5 to 10 km across strike (Fig. 2*A*) argue for advection-related cooling versus thrust heating.

Although the modeling process does not allow for a statistical evaluation of potential variations in geometry or kinematics of the palinspastic reconstruction on modeled dates, the thermochronologic data for samples at the surface cover the three main lithotectonic belts of the region and broadly agree with the modeled dates produced by Pecube-D (Fig. 2). All AHe and AFT data fall within uncertainty of their modeled dates across the surface, indicating that the low-temperature history of this region (i.e., the process of exhumation from ~110 °C to surface conditions at present time) is well explained by the Move palinspastic reconstruction. The ZHe data overlap the modeled dates within uncertainty in the northern part of the ophiolite, but farther south in the ophiolite and metamorphic belts the measured ZHe dates are generally younger than the modeled dates (e.g., observed dates of ~2 Ma vs. modeled dates of ~3 Ma). However, this discrepancy is small relative to uncertainty and is highly sensitive to the spacing of steps in the *Move 2D* model.

The plot of cooling date versus distance on Fig. 2*A* shows that exhumation in the Central Range was variable in space and time and can be interpreted as distinct kinematic events. The data presented here capture cooling of 1) the overlying ophiolite associated with duplexing of underlying Ruffaer metamorphic belt (125 to 150 km) from 16 to 10 Ma; 2) uplift and exhumation associated with emplacement of the ophiolite and repeated Ruffaer metamorphic rocks over the Australia margin from 10 to 8 Ma (ZHe dates between 125 and 150 km) (Fig. 2); and 3) uplift driven by reactivation of basement faults (57) starting at 6 Ma in the south (25 to 100 km), 4 Ma in the north (100 to 150 km), and the final uplift and exhumation along the southern basement fault (50 to 125 km) from 3 Ma to present day (*SI Appendix*, Fig. S2 and Movies S1 and S2). Measured ZHe, AFT, and AHe dates in the metamorphic belt that cluster between 1 and 2 Ma all require rapid exhumation at this time. In contrast, ZHe, AFT, and AHe dates from the ophiolite are separated in time by 2 to 5 My. The Move and Pecube modeling indicates that these clusters of thermochronometer dates in the ophiolite are explained by discrete kinematic events (*SI Appendix*, Fig. S2 and Movies S1 and S2). For example, in the ophiolite, the pattern of AHe dates (~130 to 145 km along the transect) was caused by thrust fault replication (duplex structure development) and associated cooling in the subsurface metamorphic rocks from 11 to 10 Ma. Later uplift and erosion that exhumed the ophiolite to the surface at 3 Ma is recorded by AHe dates of 3 to 7 Ma (Fig. 2 and Dataset S1). In the metamorphic belt, the 1 to 2 Ma ZHe, AFT, and AHe dates record cooling and exhumation after basement thrust reactivation beneath this zone from 4 to 3 Ma and continued uplift from motion on basement faults to the south (*SI Appendix*, Fig. S2 and Movies S1 and S2). In the fold-thrust belt, the 2 to 3 Ma AFT dates were locked in during motion on the southernmost basement thrust from 6 to 4 Ma and 3 Ma to present.

Erosion was calculated via two separate methods. For the ophiolite, the original surface of the ophiolitic unit was tracked throughout the palinspastic reconstruction, allowing a direct calculation of the difference between the original and current surface. This subtracted difference provides the cumulative ophiolite eroded through each model step. The total erosion of all lithologic units along the cross-section was calculated using the grid of points and topographic surface. For each step, the points nearest the current surface were tracked to the following step. The vertical difference between these points reflects the amount of total erosion in that step. To avoid minor errors in drafting and errors associated with grid spacing, this erosion calculation was restricted to points above 100 m in elevation.

### GEOCLIM Weathering Model.

The weathering component of GEOCLIM, called DynSoil, represents a 1D vertical weathering profile (regolith). The model computes physical erosion, regolith production at the bedrock/regolith interface, and chemical weathering of silicate minerals within the profile. We used the steady-state version of DynSoil, following Park et al. (9). Dynsoil is conceptually similar to a vertical reactive-transport model. As such, it has two end-member regimes: the supply-limited regime, where weathering rate is determined by erosion rate, and the kinetically limited regime, where weathering rate is determined by climate conditions. *SI Appendix*, Fig. S3 shows a schematic representation of the model. The three main equations of the steady-state weathering model are

$$E=kq^{0.5}s,$$

with *E* being the erosion rate, *q* the runoff rate, and *s* the topographic slope.

$$\begin{array}{c} h=d_{o}\log\left(k_{\mathrm{rp}}qexp\left(\frac{-\;E_{A}}{R}\left(\frac{1}{T}-\frac{1}{T_{o}}\right)\right)/E\right), \end{array}$$

with *h* being the regolith thickness and *T* the surface temperature.

$$\begin{array}{c} W=\chi_{\mathrm{CaMg}}E\left(1-exp\left(-k_{d}\left(1-exp\left(\;-\;k_{w}q\right)\right)exp\left(\frac{-\;E_{A}}{R}\left(\frac{1}{T}-\frac{1}{T_{o}}\right)\right)\frac{{\left(h/E\right)}^{\sigma +1}}{\sigma +1}\right)\right), \end{array}$$

where *W* is the weathering rate (in mol/m^2^/y). The model parameters are *R*, the universal gas constant; *E_A_*, the apparent activation energy at the reference temperature *T_o_*; *σ*, an exponent controlling the sensitivity of weathering reaction rate to regolith age; *k_w_*, the runoff sensitivity constant; *k_d_*, the weathering reaction rate proportionality constant; *χ_CaMg_*, the concentration of Ca and Mg in bedrock; *k_rp_*, the regolith production proportionality constant; *d_o_*, the characteristic decay depth of regolith production; and *k_e_*, the erosion proportionality constant.

*W* is calculated for five lithological classes of silicate rocks: felsic, intermediate, mafic, metamorphic, and siliclastic sediments, each of them having different amounts of Ca-Mg (*χ_CaMg_*), the other parameters being the same for all the classes of rocks. Ultramafic rocks, which may increase the climate impacts of this erosion and weathering, are not explicitly included because they are part of the “mafic” class in the lithology database of Hartmann and Moosdorf (84) that is used in GEOCLIM. See Park et al. (9) for a more complete description of the model and its parameters.

In this contribution, we used the runoff and temperature fields from ERA5 reanalysis (44), the slope field was computed from Shuttle Radar Topography Mission (SRTM) digital elevation model (85) at 30” of resolution, and interpolated at 30′ (0.5°) of resolution, and the lithological map is from Hartmann and Moosdorf (84). The weathering model was run solely on New Guinea. *SI Appendix*, Fig. S4 shows the maps of the boundary conditions (temperature, runoff, and slope) as well as the computed erosion rate. Because erosion rate in GEOCLIM is proportional to slope, we chose to alter the slope field in GEOCLIM to mimic the temporal evolution of erosion flux predicted by MOVE 2D. At each time step, we uniformly multiplied the modern slope field by the MOVE 2D erosion flux of the current time step divided by the MOVE 2D erosion flux at the final 0.5 to 0 Ma step (modern erosion). This ensures that the modern (0.5 to 0 Ma) GEOCLIM erosion flux corresponds to modern conditions and that the evolution of GEOCLIM erosion flux relative to its modern value reflects the evolution of MOVE 2D erosion flux relative to its modern value.

To compare erosion estimates from Pecube and GEOCLIM, the cumulative cross-sectional area eroded from the palinspastic reconstruction (Fig. 4*A*) was converted to the volumetric erosion rate by assuming the calculations for our transect are applicable across the E-W extent of the Central Range. The two calculations yielded similar results.

The silicate weathering feedback keeps the carbon cycle balanced, meaning that any local change in silicate weathering flux is compensated by a change in atmospheric CO_2_ (and then global mean temperature), ultimately resulting in zero net change of global weathering flux. All else being equal, a higher atmospheric CO_2_ concentration generates higher global silicate weathering flux, because of warmer temperature and enhanced runoff (7). This is why a CO_2_ drawdown, and associated global cooling, reduces the global weathering flux, compensating for a local weathering increase. Therefore, to estimate the scaling between the local weathering increase due to the rise of New Guinea, one must estimate the weathering flux sensitivity to global warming induced by CO_2_ increases, and conversely, how much global cooling decreases the weathering flux. Following Park et al. (9) we estimated the weathering sensitivity to temperature by performing additional GEOCLIM simulations at the global scale, with climate fields taken from the GFDL CM2.0 climate model simulations (49) at several CO_2_ levels and all else held constant. These experiments were repeated using CESM 1.2.2.1, with a slab ocean model (50).

The first set of simulations (GEOCLIM-GFDL) shows an increase of global silicate weathering flux by ~0.26 Tmol/y per °C of global warming due to higher CO_2_. This relationship is linear (n = 3) up to four times preindustrial CO_2_ (*SI Appendix*, Fig. S5), which is the highest available CO_2_ level in this set of climate simulations. The second set of simulations (GEOCLIM-CESM) shows a nonlinear increase of global weathering, from 0.27 Tmol/y/°C in a cool climate (0.75 times preindustrial CO_2_, that is ~−1 °C) to 0.56 Tmol/y/°C in a warm climate (three times preindustrial CO_2_, that is ~+5 °C). We have anchored the curves at the maximum temperature difference from today to the Late Miocene of 6 °C (Fig. 4) to show the maximum difference in weathering sensitivities of 0.26 to 0.56 Tmol/y/°C (*SI Appendix*, Fig. S5).

*SI Appendix*, Fig. S6 shows the depletion of weathering profiles, that is, the fraction of remaining cations left in silicate minerals being eroded, computed by GEOCLIM at several time steps. This plot allows visual quantification, throughout the exhumation history, of how close a given location is to the kinetically limited and supply-limited modes. At 6.8 to 6.1 Ma, the highest erosion flux in the reconstruction, most of the orogen has less than 20% of cations depleted, while it is closer to 40% under modern conditions. This is characteristic of the kinetically limited regime and explains why the twofold increase of erosion flux from 8 Ma to 6.5 Ma has a muted impact on weathering rate (Fig. 4*B*).

## Supplementary Material

Appendix 01 (PDF)

Dataset S01 (XLSX)

Dataset S02 (XLSX)

Dataset S03 (XLSX)

Movie S1. Step-by-step 0-300 km width reconstruction of the New Guinea arc-continent collision. The top panel shows predicted surface thermochronometer dates for AHe, AFT, and ZHe. The lower panel shows the thermal field and Move 2D lines. The floor of modern basins are depicted in orange to highlight timing of formation and extent through time. Green shows original surface of ophiolite.

Movie S2. Step-by-step 0-600 km width reconstruction of the New Guinea arc-continent collision. The top panel shows predicted surface thermochronometer dates for AHe, AFT, and ZHe. The lower panel shows the thermal field and Move 2D lines. The floor of modern basins are depicted in orange to highlight timing of formation and extent through time. Green shows original surface of ophiolite.

## Data Availability

All study data are included in the article and/or supporting information. Data are available in Datasets S1–S3. Code and additional supporting files are available at https://doi.org/10.5061/dryad.p2ngf1vx3 (86).
