# Supplementary material for: The rise of New Guinea and the fall of Neogene global temperatures
Source: Proc Natl Acad Sci U S A. 2023 Sep 25;120(40):e2306492120. doi: 10.1073/pnas.2306492120 (PMC10556579; doi:10.1073/pnas.2306492120)
Supplement: Supplementary file 1 — Appendix 01 (PDF) [file pnas.2306492120.sapp.pdf]

PNAS

Supporting Information for

The rise of New Guinea and the fall of Neogene global temperatures

Peter E. Martin[1], Francis A. Macdonald[2]*, Nadine McQuarrie[3], Rebecca M. Flowers[1], Pierre J.Y. Maffre[4,5]

[1]Department of Geological Sciences, University of Colorado Boulder; Boulder, CO 80309, USA.

[2]Department of Earth Science, University of California, Santa Barbara; Santa Barbara, CA 93106, USA.

[3]Department of Geology and Environmental Science, University of Pittsburgh, Pittsburgh, PA 15260, USA.

[4]Department of Earth and Planetary Science, University of California, Berkeley 94720, CA, USA.

[5]Aix-Marseille Université, CNRS, IRD, INRAE, Collège de France, CEREGE, Aix-en-Provence, France.

Corresponding author: Francis A. Macdonald

Email: francism@ucsb.edu

This PDF file includes:

Figs. S1 to S6
Tables S1 to S4
Captions for Movies S1 to S2
Captions for Data S1 to S3

Other supporting materials for this manuscript include the following:

Movies S1 to S2
Data S1 to S3 [(U-Th)/He data; Zircon U-Pb data; Geo- and thermochronology reduced data]

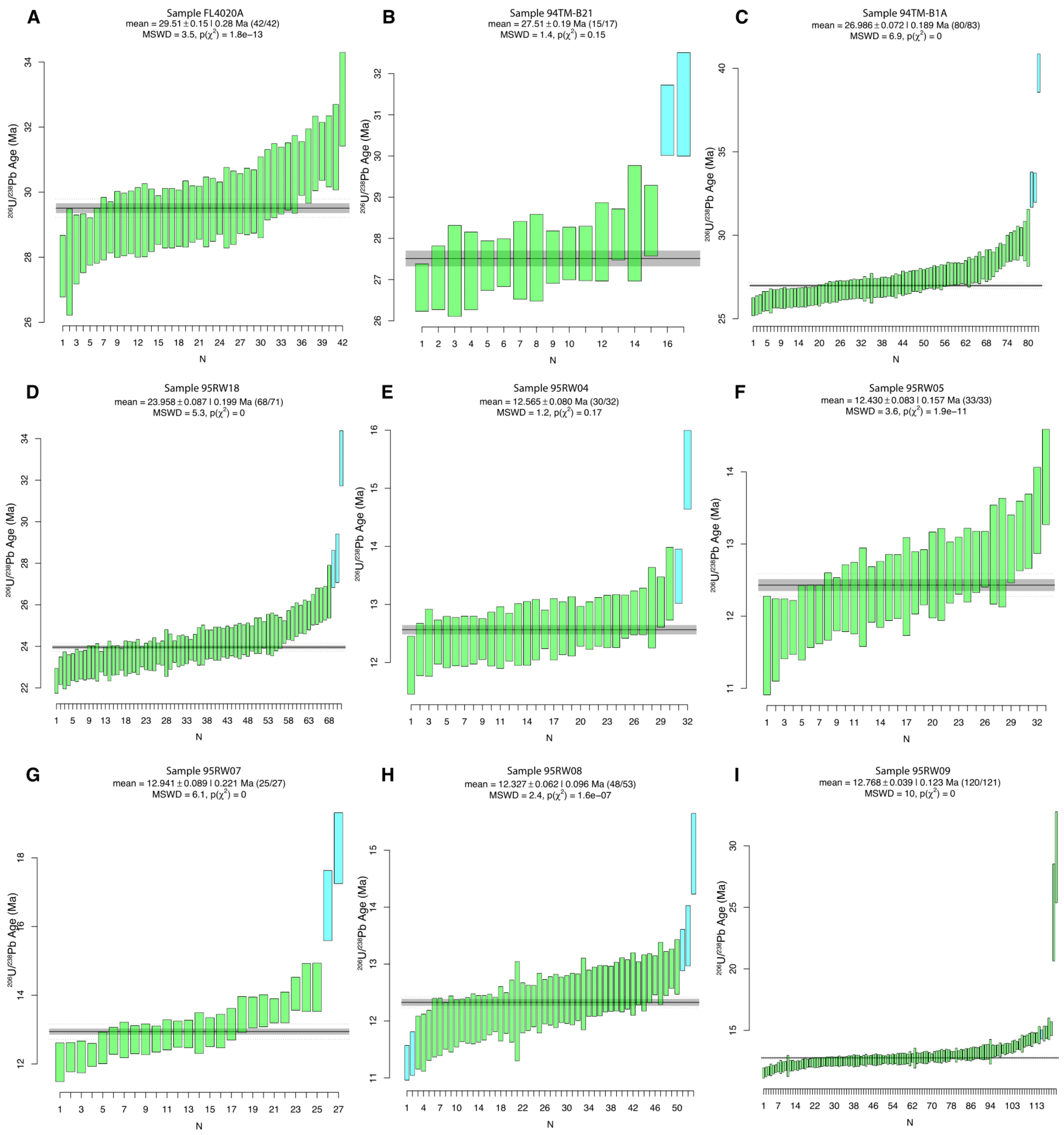

Fig. S1. Weighted mean and outlier U-Pb analyses, generated with IsoplotR (67).

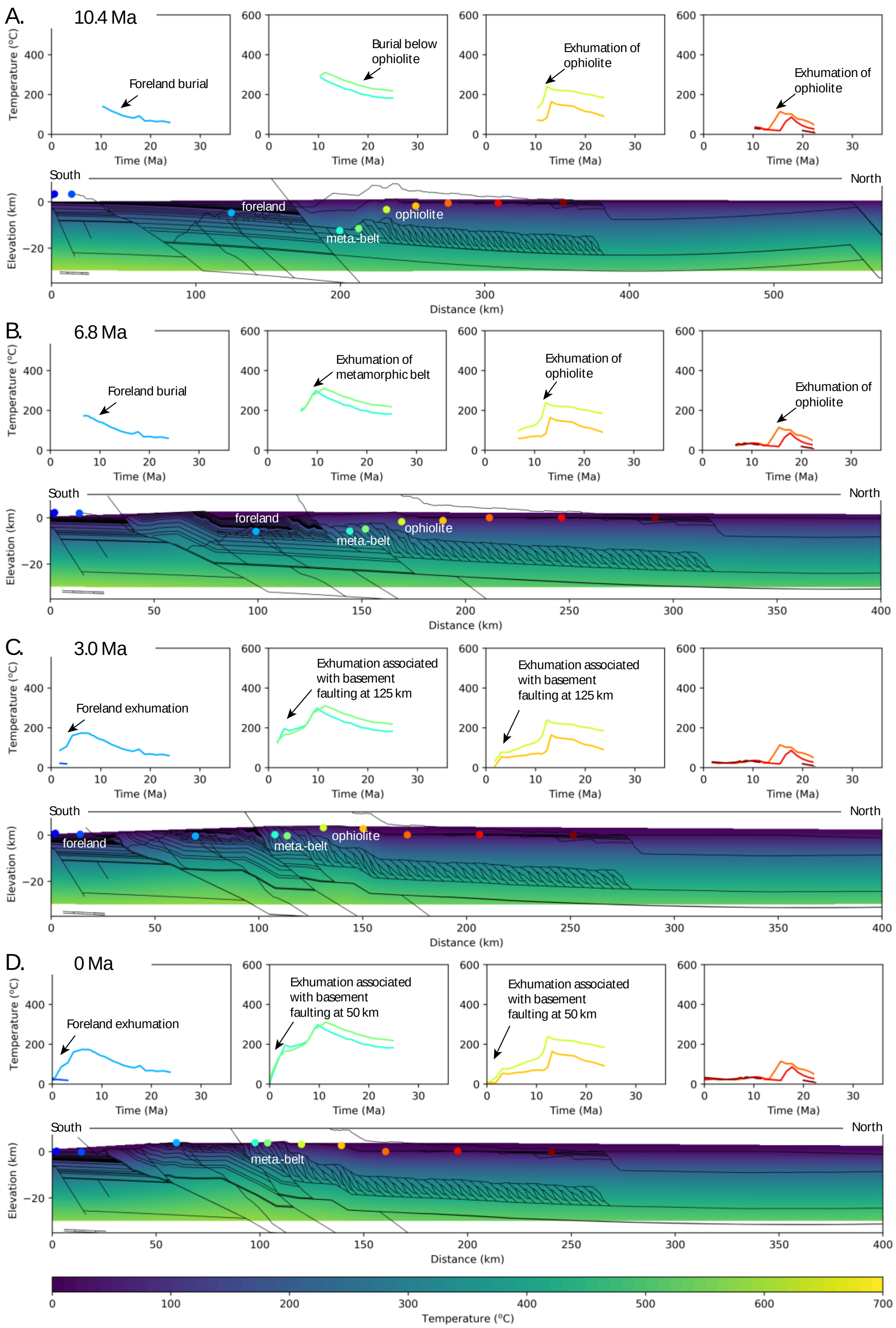
A.
10.4 Ma
Foreland burial
Burial below ophiolite
Exhumation of ophiolite
Exhumation of ophiolite
Temperature (°C)
Time (Ma)
South
North
foreland
ophiolite
meta-belt
Elevation (km)
Distance (km)
B.
6.8 Ma
Foreland burial
Exhumation of metamorphic belt
Exhumation of ophiolite
Exhumation of ophiolite
C.
3.0 Ma
Foreland exhumation
Exhumation associated with basement faulting at 125 km
Exhumation associated with basement faulting at 125 km
D.
0 Ma
Foreland exhumation
Exhumation associated with basement faulting at 50 km
Exhumation associated with basement faulting at 50 km
Temperature (°C)

Fig. S2. Snapshots of Pecube model with Temperature-time paths. Temperature scale for each snapshot is shown at the bottom of D. Model results from a surface heat production of 3.0 µWm^3 and e-folding depth of 20 km; meta.-belt = metamorphic belt. A) 10.4 Ma as exhumation begins in the preserved ophiolite belt. B) 6.8 Ma as exhumation of the ophiolite begins above the preserved metamorphic belt. C) 3.0 Ma as thrusting migrates inboard with increased coupling with the continent. D) 0 Ma. For C and D, we only model the Central Range fold and thrust belt and do not not account for effects from the accretion of the Finisterre block to the northeast, recent magmatism, or the development of a strike slip margin to the north.

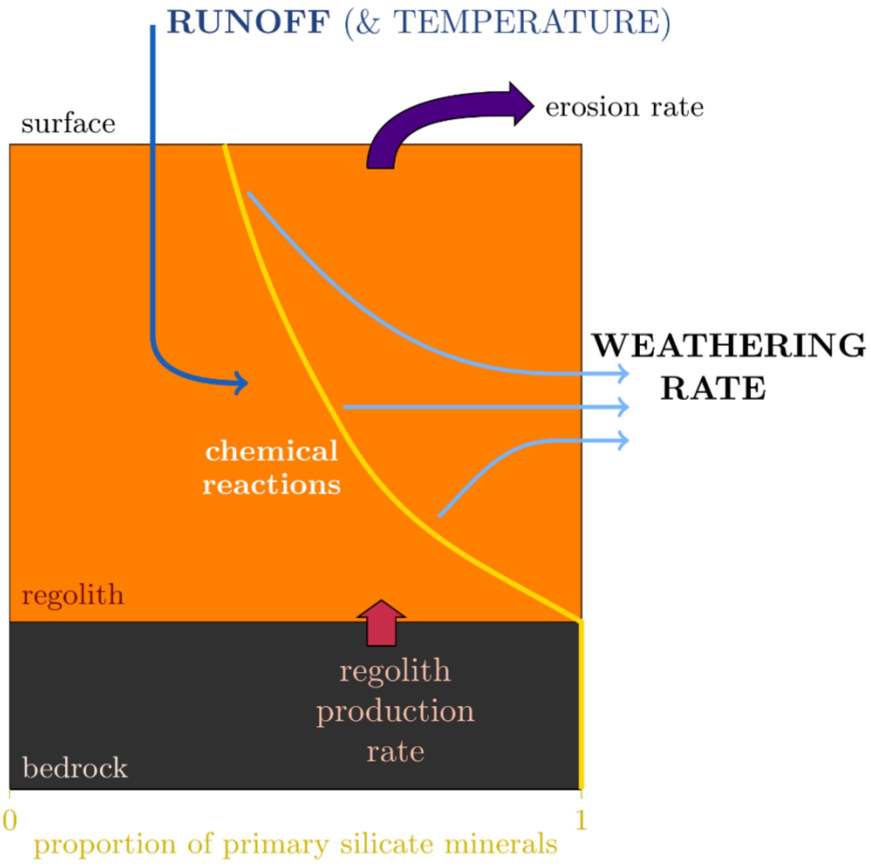

Fig. S3. Schematic representation of the weathering mode DynSoil. The proportion of primary minerals decreases towards the surface because of weathering reactions. An equilibrium profile is determined by a trade-off between regolith production rate (equal to erosion rate, at steady-state) and weathering reaction rates.

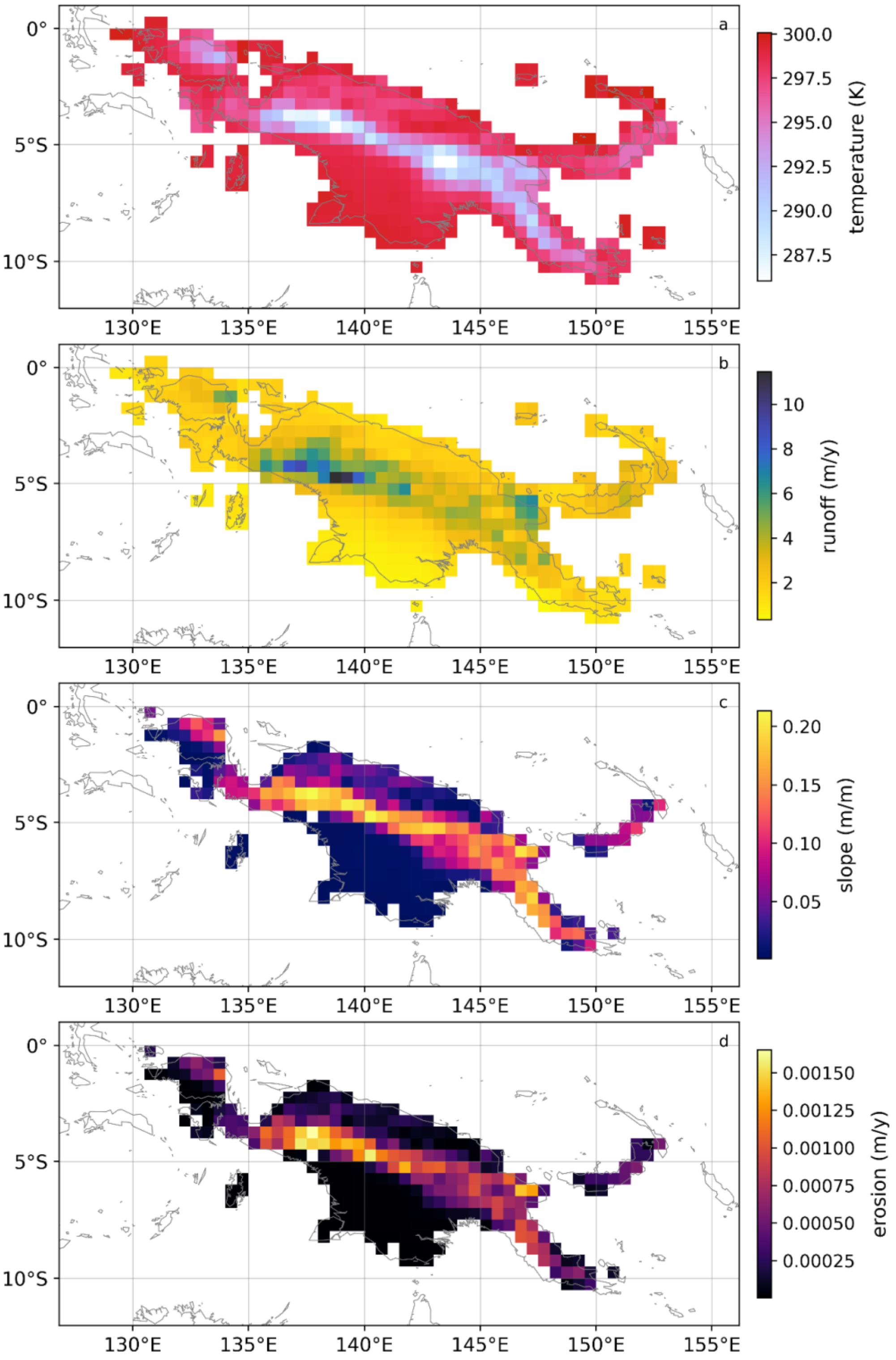

Fig. S4. Maps of temperature (A), runoff rate (B), topographic slope (C) used in the GEOCLIM model, and the computed erosion rate (D). All fields are at 0.5° resolution with modern slope and erosion fields.

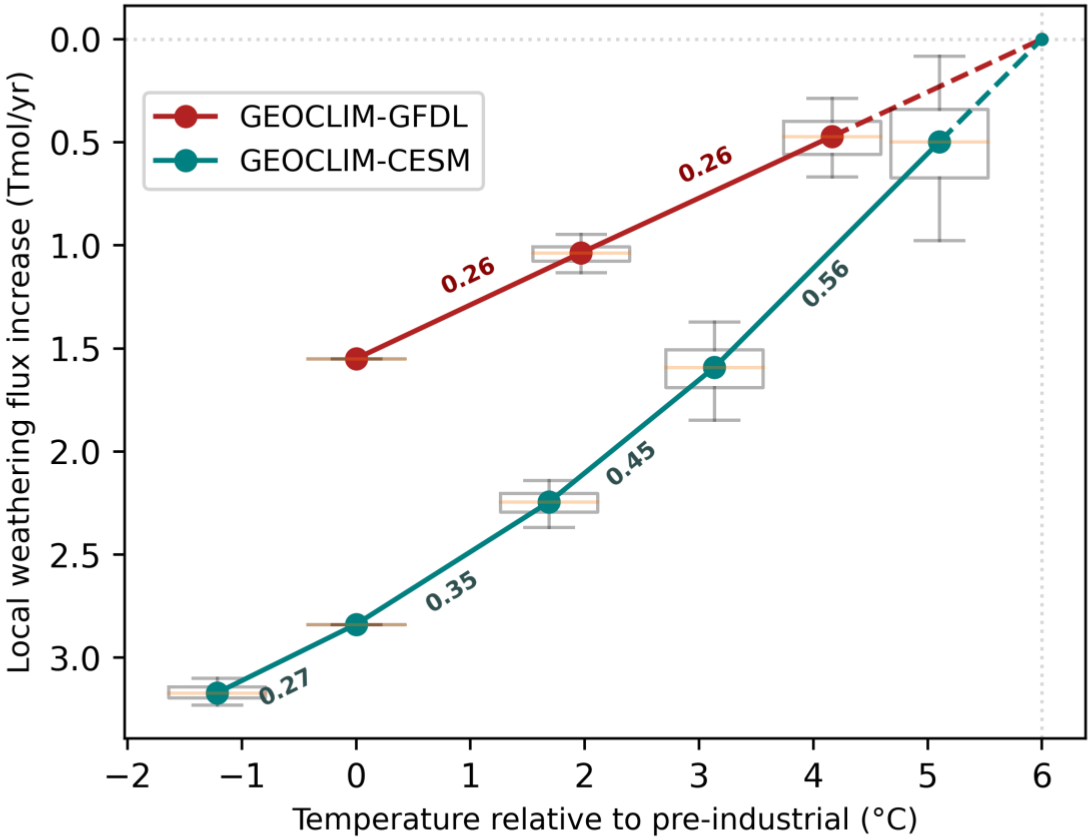

Fig. S5. Relationship between local silicate weathering flux anomaly and its equivalent global mean surface temperature anomaly calculated with respect to the pre-industrial simulation. The curves were anchored at +6°C, which was chosen as maximum temperature difference from the Late Miocene to pre-industrial temperatures (Fig. 4C). The curves were extrapolated up to +6°C (dashed lines) as no climate simulation was available at such high temperature. This figure can be interpreted as the relation between a local weathering increase since the Late Miocene and global cooling towards modern temperature. The temperature-weathering relationship is estimated with global scale GEOCLIM simulations with climate fields from GFDL CM2.0 coupled ocean-atmosphere experiments (9, 49, red curve), or CESM 1.2.2.1 slab ocean (50, teal curve). Each dot and boxplot is one GEOCLIM simulation. The boxplots show the uncertainties of global silicate weathering flux due to the different selected plausible parameter combinations (9). Note that this uncertainty collapses to 0 at pre-industrial temperature because it was chosen as the reference weathering field. The values along the curves indicate the local slope (i.e., weathering sensitivity, in Tmol/yr/°C).

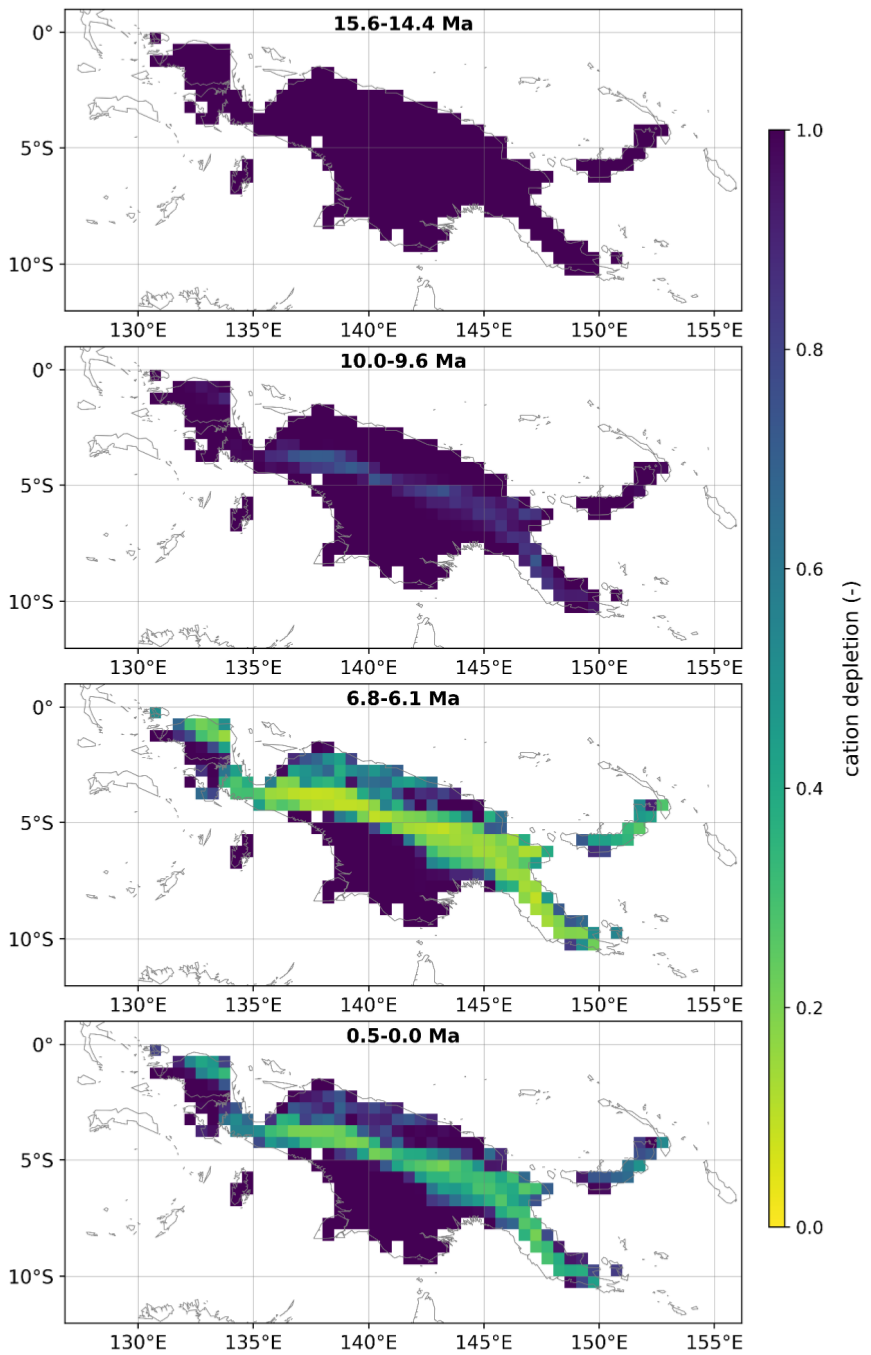

Fig. S6. Maps of cation depletion at the top of the regolith for four selected time steps. Depletion ranges from 0 (unweathered mineral) to 1 (fully depleted minerals). Values close to 0 correspond to the kinetically-limited regime, whereas values close to 1 correspond to the supply-limited regime. The 6.8–6.1 Ma time step is the time when the erosion flux predicted by MOVE 2D is the highest. At this time step, most of the orogen exhibits a value <0.2.

Table S1. Dated samples from the three northern transects (Existing Analyses from 29).

Sample Name	Lithology	Transect	Existing Analyses*	New Analyses	Latitude	Longitude
95RW04	Diorite	Western	AFT	AHe, ZHe, U-Pb	-3.19967	136.653167
95RW05	Diorite	Western	AFT	AHe, ZHe, U-Pb	-3.22267	136.653167
95RW07	Monzodiorite	Western	AFT	AHe, ZHe, U-Pb	-3.217	136.653333
95RW08	Monzodiorite	Western	AFT	AHe, ZHe, U-Pb	-3.212	136.656667
95RW09	Monzodiorite	Western	AFT	ZHe, U-Pb	-3.208	136.657667
95RW15	Salte	Western	AFT	AHe	-3.39617	136.6615
95RW21	Peridotite	Western		AHe, ZHe	-3.26683	136.616333
95RW31A	Sandstone	Western	AFT		-3.38833	136.667167
95RW31B	Red siltstone	Western	AFT	ZHe	-3.38833	136.667167
94RW21	Hornfelsed sandstone	Central	AFT	AHe, ZHe	-3.7305	137.4445
94RW23	Slate	Central	AFT		-3.7605	137.463667
94RW30	Phyllite	Central		ZHe	-3.67667	137.402167
95RW38	Phyllite with carbonate	Central	AFT		-3.64217	137.362667
95RW39	Phyllite	Central	AFT	ZHe	-3.63983	137.359833
95RW45	Microdiorite	Central		AHe, ZHe	-3.49833	137.286
93RW16A	Diorite	Eastern	AFT	AHe	-3.42167	138.57
93RW16B	Quartz-plagioclase vein	Eastern	AFT	AHe	-3.42167	138.57
93RW17	Muscovite	Eastern	AFT	AHe	-3.42333	138.573333
93RW18	Tonalite	Eastern	AFT	AHe, ZHe, U-Pb	-3.345	138.66
93RW19	Quartz diorite	Eastern	AFT	AHe, ZHe	-3.34833	138.661667
94TM-DB1A	Quartz diorite	Eastern	AFT	AHe, ZHe, U-Pb	-3.3695	138.619
95RW61	Phyllite	Eastern	AFT		-3.50767	138.507667
95RW62	Silty phyllite	Eastern	AFT	ZHe	-3.51733	138.517333

*A subset of these samples were also used for zircon fission track analyses, but largely do not pass the chi-squared test; these analyses are therefore not included in this study

Table S2. Dated samples from the southern transect (from 19, 29).

Sample Name	Lithology	Existing Analyses	Latitude	Longitude
91-RW-TNG-1	Quartz diorite	AFT	-4.04806	137.1025
91-RW-TNG-2	Quartz diorite	AFT	-4.05778	137.108611
91-RW-TP	Trachydacite	AFT	-4.06694	137.108611
90-TM-CORDI-1	Trachydacite	AFT	-4.06944	137.11
91-AQ-CZW-1	Trachydacite	AFT	-4.07833	137.112778
K4-197-91	Dacite	AFT	-4.08778	137.108056
ET-69/70-91	Quartz monzonite	AFT	-4.09	137.113611
90-TM-GBT-2	Granodiorite	AFT	-4.09556	137.128333
90-TM-TEMB-1	Sandstone	AFT	-4.15556	137.090556
91-MC-T1	Altered intrusive	AFT	-4.15944	137.086667
90-TM-11.3-1	Siltstone	AFT	-4.16222	137.084444
91-MC-T2	Altered intrusive	AFT	-4.17	137.095556
90-TM-13.6-3	Sandstone	AFT	-4.17083	137.098056
90-TM-16.9-1	Sandstone	AFT	-4.19028	137.090278
90-TM-17.6-2	Sandstone	AFT	-4.19611	137.089444
90-TM-18.8-3	Sandstone	AFT	-4.20306	137.084444
90-TM-19.9-3	Sandstone	AFT	-4.20889	137.080833

Table S3. Input parameters used for Move (kinematic) and Pecube (thermal) modeling.

**Material Properties**	
Effective elastic thickness (km)	70-75
Crustal density (kg m-3)	2600
Mantle density (kg m-3)	3300
Specific heat capacity (J kg-1 K)	800
Crustal volumetric heat production (μW m-3)	2.0-4.0
e-folding depth (km)	15-20
Thermal conductivity (W m-1 K)	2.5
**Numerical properties**	
Temperature at base of model (°C)	1300
Model thickness (km)	110
Surface temperature (°C)	24
Atmospheric lapse rate (°C km-1)	6.5
Kinematic grid spacing (km)	0.5
Horizontal node spacing (km)	0.5
Vertical node spacing (km)	1
Model domain (km)	820 x 110 x 5
Model start time (Ma)	60

Table S4. Shortening amount, age, and velocity of fault bound blocks in palinspastic reconstruction; OOS = out of sequence. Shortening amounts were iteratively adjusted to reproduce the balanced cross-section, surface geology, and topography (57). The age of displacement was iteratively adjusted to best replicate measured thermochronometers.

Structure	Shortening (km)	Initiation age (Ma)	Rate (mm/yr)
Ophiolite	220	35.0	15.0
Metamorphic duplex	108	20.4	15.0
Metamorphic duplex	71	13.2	21.7
Fold thrust belt	53	9.5	18.6
Fold thrust belt	13	6.8	8.4
Basement fault south	18	6.0	8.4
Basement fault north	8	3.9	8.4
Basement fault south	8	3.0	3.2
Basement fault south (OOS)	3	0.5	6.0

Movie S1 (separate file). Step-by-step 0-300 km width reconstruction of the New Guinea arc-continent collision. The top panel shows predicted surface thermochronometer dates for AHe, AFT, and ZHe. The lower panel shows the thermal field and Move 2D lines. The floor of modern basins are depicted in orange to highlight timing of formation and extent through time. Green shows original surface of ophiolite.

Movie S2 (separate file). Step-by-step 0-600 km width reconstruction of the New Guinea arc-continent collision. The top panel shows predicted surface thermochronometer dates for AHe, AFT, and ZHe. The lower panel shows the thermal field and Move 2D lines. The floor of modern basins are depicted in orange to highlight timing of formation and extent through time. Green shows original surface of ophiolite.

Dataset S1 (separate file). (U-Th)/He data.

Dataset S2 (separate file). Zircon U-Pb data.

Dataset S3 (separate file). Geo- and thermochronology data used for comparison with Pecube predicted dates.
